# Towards a Direct Consideration of Microstructure Deformation during Dynamic Recrystallisation Simulations with the Use of Coupled Random Cellular Automata—Finite Element Model

**DOI:** 10.3390/ma17174327

**Published:** 2024-08-31

**Authors:** Kacper Pawlikowski, Mateusz Sitko, Konrad Perzyński, Łukasz Madej

**Affiliations:** Faculty of Metals Engineering and Industrial Computer Science, AGH University of Krakow, al. Adama Mickiewicza 30, 30-059 Kraków, Poland; kpawliko@agh.edu.pl (K.P.); msitko@agh.edu.pl (M.S.); kperzyns@agh.edu.pl (K.P.)

**Keywords:** random cellular automata, dynamic recrystallisation, inverse analysis, Fe30Ni, parallelisation

## Abstract

Dynamic recrystallisation (DRX) is one of the fundamental phenomena in materials science, significantly impacting the microstructure and mechanical properties of components subjected to large plastic deformations. Experimental research on that topic carried out for a wide range of new metallic materials is often supported by computational materials science. A direct consideration and detailed understanding of this phenomenon are possible with a class of full-field numerical models based on the cellular automata (CA) method. However, the classical CA approach is based on a regular, fixed computational space and has limitations in capturing large deformations of the computational domain. Therefore, the main goal of the work is to develop and implement an alternative solution to overcome this limitation. The proposed solution is based on coupling the finite element (FE) method with the random cellular automata (RCA) approach. Such a model can directly consider the influence of geometrical changes in microstructure during large plastic deformation on recrystallisation progress. Details of the developed RCA DRX model assumptions and coupling issues with FE mesh are discussed. Particular attention is also paid to increasing model efficiency and robustness studies.

## 1. Introduction

Usually, novel processing technologies and innovative engineering materials are designed and developed through costly and time-consuming experimental and laboratory research that often faces investigative limitations [1]. However, recent progress in computer systems and available computation power opened new perspectives for computer-aided engineering approaches. They create new and often unrecognised opportunities for materials science applications that complement and extend experimental investigations, allowing unprecedented observations of phenomena at various length scales [2].

One of such fundamental phenomena in materials science that significantly impacts the microstructure and mechanical properties of components subjected to large plastic deformations is dynamic recrystallisation (DRX). However, when the microscale features of dynamic recrystallisation in complex microstructures are numerically investigated, appropriate capturing of the local heterogeneities becomes critical [3]. These local material interactions affect the dynamic recrystallisation and eventually result in specific macroscopic behaviour of the final product. Therefore, sophisticated numerical modelling tools like phase field [4], level set [5], vertex [6], Monte Carlo [7], or cellular automata methods [8] often must be used. 

In recent years, significant progress has been made in the development of the latter approaches, the cellular automata models explicitly tailored for dynamic recrystallisation [9,10,11,12]. The primary difficulty with DRX simulations is that the process is accompanied by substantial material deformation at elevated temperatures, leading to grain shapes and crystallographic orientation changes multiple times during a single processing stage. Capturing these spatial deformations is essential for a comprehensive understanding of the microstructural evolution, directly affecting manufactured products’ final in-use properties. 

**Figure 1:**
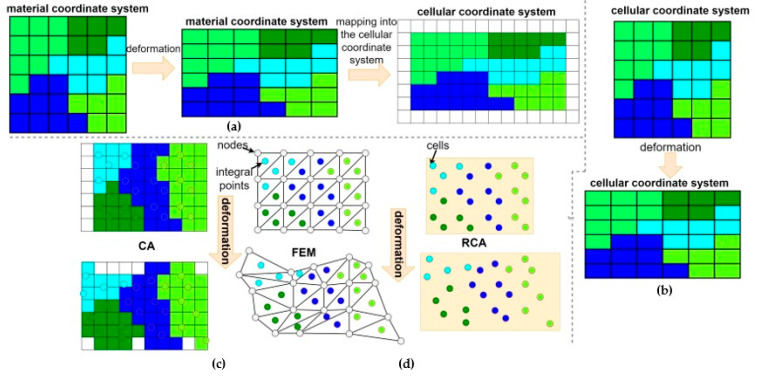
Different approaches to the CA space deformation modelling: (**a**) space mapping, (**b**) geometric changes in cells, (**c**) CAFE, and (**d**) RCAFE.

However, the classical CA method is based on a regular, fixed computational space and is limited in capturing such large deformations [13]. Therefore, recent advancements in CA-based DRX models have addressed this challenge by incorporating sophisticated deformation algorithms that enable the simulation of CA space deformation alongside microstructural evolution from simple elongation [14] to different mapping techniques [15,16] shown in Figure 1a, b. The major driving force of DRX in these models is the difference in the dislocation density. Usually, the dislocation density evolution is described by different variants of the Kocks–Mecking relation as follows [17]:

(1) $$\frac{d\rho }{d\epsilon }=k_{1}\sqrt{\rho }-k_{2}\rho ,$$

where $k_{1}=\frac{2\theta_{0}}{\alpha \mu b}$ is a constant that represents work hardening, $k_{2}=\frac{2\theta_{0}}{\sigma_{s}}$ is a softening parameter that represents recovery of dislocations, *b* is the magnitude of Burger’s vector, α is the dislocation interaction term (set to 0.5), *μ* is the shear modulus, $\theta_{0}$ is the hardening rate, and $\sigma_{s}$ is the steady-state flow.

A more advanced approach involves coupling CA models with the finite element method to account for the continuum mechanics of deformation (Figure 1c). By integrating FE with CA, researchers can simulate the spatially varying strain fields that drive DRX and accurately predict the evolution of microstructures under complex deformation conditions. Often, J2 plasticity is used in the FE part. This hybrid approach combines the advantages of both techniques, leveraging the discrete nature of CA for modelling microstructural evolution and the continuum approach of FE for handling spatial deformation. Additionally, more advanced crystal plasticity finite element models can also be used [18]. In this case, the authors proposed the scaling factor λ for the adaptation of the CA algorithm to the CPFEM solution and scale dislocation density based on the cell location as follows:

(2) $$\rho =\{\begin{array}{c} \rho ,\;for\;grain\;interior\\ \lambda \rho ,\;for\;grain\;boundary \end{array}$$

(3) $$\lambda =1-c_{3}\ln\left(\frac{{\dot{\varepsilon }}_{0}}{\dot{\varepsilon }}\right)\exp\left(\frac{T}{T_{melt}}\right)\exp\left(-c_{4}\varepsilon_{i}^{P}\right),$$

where *c*_3_ and *c*_4_ are fitting constants (*c*_3_ = 0.032, *c*_4_ = 3 [18]); $\dot{\varepsilon }$ and ${\dot{\varepsilon }}_{O}$ are the macro strain rate and reference strain rate, respectively; $T_{melt}$ is the melting temperature, and $\varepsilon_{i}^{P}$ is the plastic strain of element *i* standing for the grain boundary.

Despite such a sophisticated approach that allows direct tracking of the change in the position of cells in computational space, the regular, structured FE mesh does not guarantee a faithful representation of the deformation on the sides of the cellular automata domain. The solution to this problem is a random cellular automata (RCA) method that allows the tracking of the position of each cell independently (Figure 1d). Such an approach considers changes in the geometry and, therefore, the conceptual surfaces of the CA cells over time. Despite the advantages, both the RCA neighbourhood selection for the transition rules and the number of RCA cells used during RCAFE calculation lead to long computational times.

That is why developing a physics-based and computationally efficient RCAFE model for DRX motivated the current research.

Subsequent RCAFE DRX model development stages, starting with experimental investigation used for acquiring input data, are presented below. The austenite model alloy Fe30Ni was selected for the RCAFE model development as a case study.

## 2. Materials and Methods

### 2.1. Flow Stress Model Development for Fe30Ni

The precision of numerical simulations in metal forming processes directly relies on the accuracy of the rheological model and the definition of mechanical and thermal boundary conditions. Among these factors, developing a reliable flow stress model that characterises material behaviour under processing conditions is particularly important. Therefore, the critical challenges in simulating thermo-mechanical processes lie in evaluating rheological parameters under varying deformation conditions. That is usually performed with the use of plastometric tests realised under a set of loading and temperature conditions. However, such tests are influenced by factors like friction or deformation heating and eventually exhibit deformation inhomogeneities. These aspects affect the interpretation of the results and can be misleading when evaluating appropriate flow stress values for FE simulations. Thus, the inverse analysis technique should be used to overcome these difficulties [19].

The general procedure for the inverse analysis concept is composed of performing plastometric tests of compression, tension, or torsion and an interpretation of results of these tests with the use of a direct problem model based on the FE model combined with the optimisation task. Such an approach directly mitigates the influence of the mentioned inhomogeneities on the reliable flow stress model determination.

In this case, the direct problem model replicates the investigated plastometric test, including the geometry of dies, a sample, and the process conditions. The mathematical formulation is expressed as follows:

(4) $$F\left(\vec{x},\vec{p}\right)=\vec{d}$$

where $\vec{x}=\left\{x_{1},\ldots ,x_{l}\right\}$ is the vector of model coefficients, $\vec{p}=\left\{p_{1},\ldots ,p_{k}\right\}$ is the vector of process parameters, and $\vec{d}=\left\{d_{1},\ldots ,d_{q}\right\}$ is the vector of the evaluated model output parameters.

**Figure 2:**
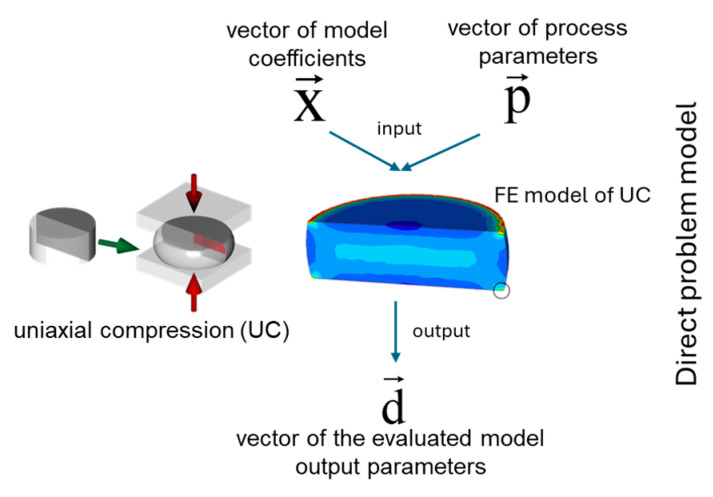
Concept of the direct problem model definition based on the uniaxial compression (UC) test.

The $\vec{x}$ vector can incorporate flow stress model coefficients, friction coefficients, heat transfer coefficients, etc. The $\vec{p}$ vector can incorporate information on the deformation degree, strain rate, temperature of the sample, temperature of the surrounding medium, etc. For the direct problem model, the $\vec{x}$ and $\vec{p}$ vectors are known. Finally, the $\vec{d}$ vector can contain the results from the model *F*, e.g., calculated forces, final sample shape, temperature field, etc. The vector $\vec{d}$ is unknown prior to the simulations, as seen in Figure 2.

However, if the $\vec{d}$ vector is known, e.g., from an experimental investigation, and the $\vec{x}$ or $\vec{p}$ is unknown, then the inverse problem can be defined. Thus, the goal of the inverse analysis is to evaluate optimal values of parameters of the $\vec{x}$ or $\vec{p}$ vectors that minimise the defined goal function as follows:

(5) $$\emptyset \left(\vec{x}\right)=\overset{n}{\underset{i=1}{\sum }}\beta_{i}{\left[{\vec{d}}_{i}^{c}\left(\vec{x},{\vec{p}}_{i}\right)-{\vec{d}}_{i}^{m}\right]}^{2}$$

where ${\vec{d}}^{m}=\left\{d_{1}^{m},d_{2}^{m},\ldots ,d_{q}^{m}\right\}$ and ${\vec{d}}^{c}=\left\{d_{1}^{c},d_{2}^{c},\ldots ,d_{n}^{c}\right\}$ are the vectors of the measured and calculated parameters, respectively; *β_i_* refers to the weights (*i* = 1 … *n*); and *n* is the number of measuring points. The measured values gathered in the vector ${\vec{d}}^{m}$ are evaluated experimentally and those in ${\vec{d}}^{c}$ are calculated by the finite element simulation.

It is shown in a number of scientific publications [20,21] that the application of the inverse analysis to the interpretation of plastometric test results for the identification of $\vec{x}$ minimises the influence of process disturbances and allows flow stress to be determined independent of the testing method and the applied stress state.

**Figure 3:**
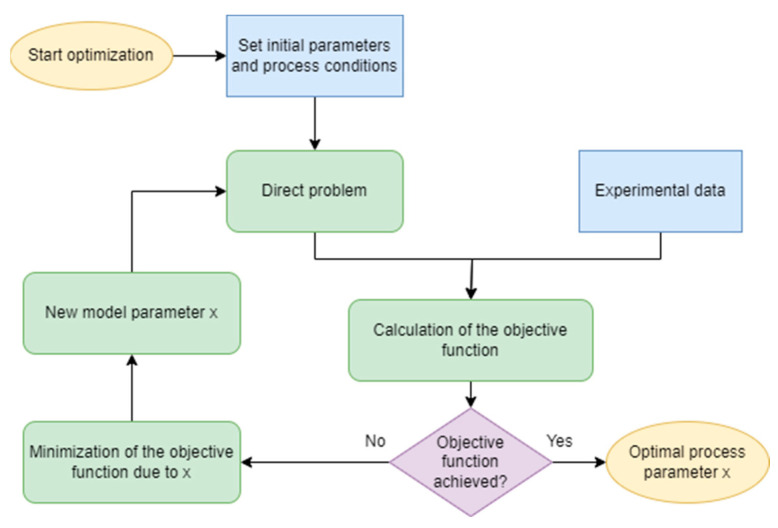
Inverse analysis flow chart.

Therefore, the practical application of the inverse analysis procedure in the current research involves the following three major steps, as shown in Figure 3:

- Acquiring experimental results (load-displacement data) from a series of tests (uniaxial compression) realised under a combination of different strain rates and temperature conditions with the use of a Gleeble 3800 (Dynamic Systems Inc., Poestenkill, NY, USA) thermo-mechanical simulator;
- Development of the direct problem model of the UC compression on the basis of the in-house finite element model [22];
- Application of the Nelder–Mead optimisation algorithm to minimisation of the goal function, which is defined as

(6) $$\emptyset \left(\vec{x}\right)=\sqrt{\frac{1}{Npt}\overset{Npt}{\underset{i=1}{\sum }}\left[\frac{1}{Nps}\overset{Nps}{\underset{j=1}{\sum }}{\left(\frac{F_{cji}\left(\vec{x},{\vec{p}}_{i}\right)-F_{mji}}{F_{mji}}\right)}^{2}\right]}$$

where *F_mij_* and *F_cij_* are the measured and calculated loads, *Npt* is the number of UC tests, and *Nps* is the number of load measurements in one test.

The minimisation of the goal function is realised with respect to the *β* coefficient in the initial flow stress equation [23] as follows:

(7) $$\sigma_{p}=\beta \frac{F_{m}}{S}\left[\frac{\dot{\varepsilon }\exp\left(\frac{Q}{RT}\right)}{{\dot{\varepsilon }}_{n}\exp\left(\frac{Q}{RT_{n}}\right)}\right]$$

where *s_p_* is the flow stress, *F_m_* is the measured load, *S* is the current sample surface value, *Q* is the activation energy, *T* is the current temperature, *T_n_* is the nominal test temperature, $\dot{\varepsilon }$ is the current strain rate, ${\dot{\varepsilon }}_{n}$ is the nominal test strain rate, and *m* is the strain rate exponent. The activation energy and strain rate exponent values are calculated based on experimentally measured loads.

Therefore, flow stress data obtained from the inverse analysis considers the local material behaviour and is insensitive to various disturbances occurring in the sample during a single deformation test. Thus, reliable flow stress data will be obtained and used to develop a flow stress equation that is valid for a range of temperatures and strain rates for further RCAFE model development.

#### 2.1.1. Uniaxial Compression Experiments

As mentioned, the austenite model alloy in the form of Fe30Ni was selected for the investigation. The detailed chemical composition is summarised in Table 1.

**Figure 4:**
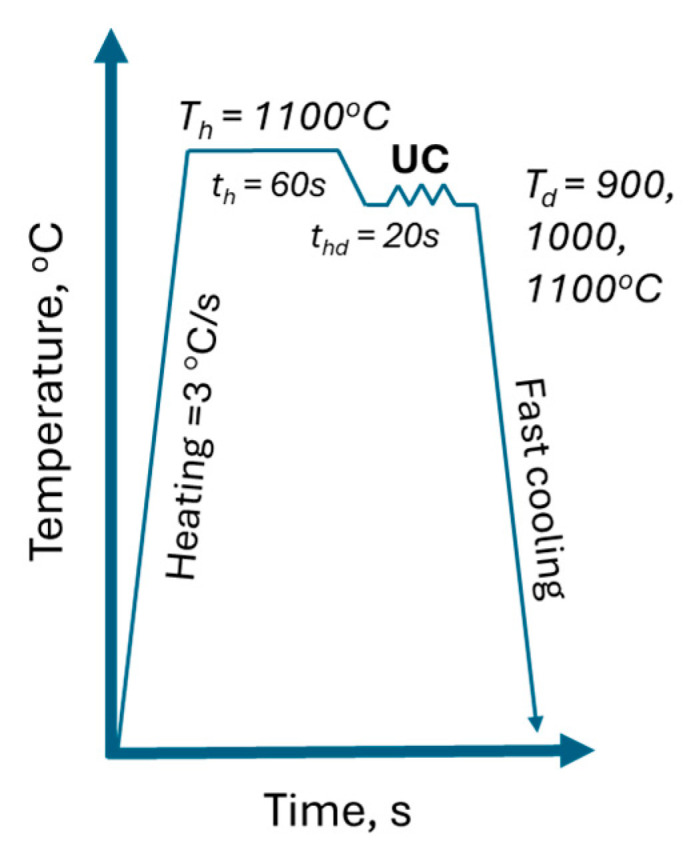
Uniaxial compression experimental setup.

The uniaxial compression tests were realised with the Gleeble 3800 thermomechanical simulator and involved deformation under a set of three different temperature and strain rate conditions: 900, 1000, and 1100 °C and 0.1, 1, and 10 s^−1^. The designed UC experimental workflow (Figure 4) ensures the homogenisation of the process conditions.

**Figure 5:**
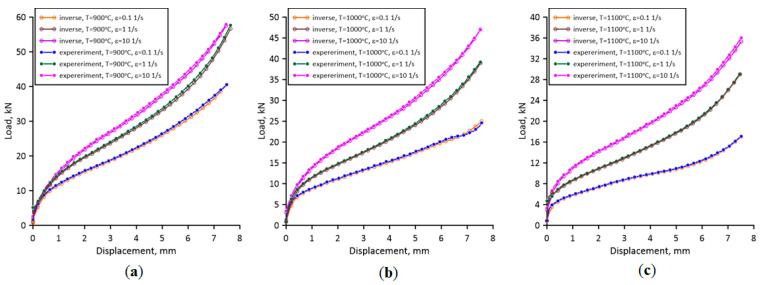
Final agreement between the measured and calculated load-displacement values after the inverse analysis at (**a**) 900 °C, (**b**) 1000 °C, and (**c**) 1100 °C.

Preheating at temperature *T_h_* aims to homogenise the microstructure and ensure a uniform temperature at the beginning of the test. During the test, a sample is compressed between two dies while forces and die displacements are recorded online, as seen in Figure 5. The recorded load-displacement data were then used as the input for the inverse analysis algorithm.

#### 2.1.2. Inverse Analysis

First, the values of *Q* and *m* were determined for the inverse analysis based on regression analysis of measured load values during the experiments. The *Q* and *m* values in Equation (4) vary in the range of 20,192–33,880 J/mol and 0.078–0.151, respectively, for the investigated process conditions. The determined values were used during the inverse analysis with the goal functions defined as (3). The direct problem model was developed using in-house FE software version 2.0 based on flow formulation. The final agreement between the experimentally measured and calculated load-displacement values after Nelder–Mead optimisation is presented in Figure 5.

**Figure 6:**
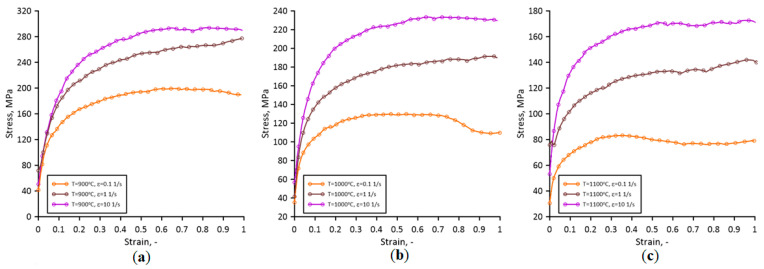
Stress–strain curves after the inverse analysis at (**a**) 900 °C, (**b**)1000 °C, and (**c**) 1100 °C.

As seen in Figure 5, the calculated load values agree very well with the experimental measurements. Therefore, the determined flow stress data are gathered in Figure 6.

The developed flow stress model of Fe30Ni was then used at the RCAFE model development stage, which is presented in the following parts of the work.

### 2.2. Efficient Random Cellular Automata Grain Growth Algorithm

As mentioned, for the random cellular automata method, the major obstacle in terms of performance is the step involved in determining the neighbours of the currently analysed RCA cell. Since the RCA space is constantly changing during the simulation, it is impossible to determine the neighbours of each cell only once during the initial step of the simulation. This operation must be repeated at each time step until the end of the simulation, affecting the computing times.

Therefore, the authors have investigated various approaches to speed up the neighbour determination procedure for these models. One of the solutions is based on associative lists [24] during neighbour identification; however, this approach depends on the selected neighbourhood radius’s physical size. A solution to this type of overhead is, for example, to use mechanisms for pre-sorting and grouping cells into containers, which were investigated in earlier research [25]. A simple benchmark was proposed to compare different developed solutions. Firstly, a random position for a precise number of points increased linearly from 1000 to 1,000,000 were distributed in a particular space. Different methods were computed based on exactly the same initial point distributions for the particular number of cells in space. In each scenario, exactly the same number of nucleons were randomly assigned at the beginning of the simulation. In each scenario, neighbours lists were computed multiple times at the beginning of each iteration, and in all scenarios, exactly 16 neighbours were chosen to simulate unconstrained grain growth. Based on the selected neighbours, the dominant ID in the neighbourhood was selected, and grain growth continued to fill the entire space. For each method and cell number, the simulation setup was computed at least three times, and after that, the average computation time was calculated. All procedure was automated via Python scripts.

**Figure 7:**
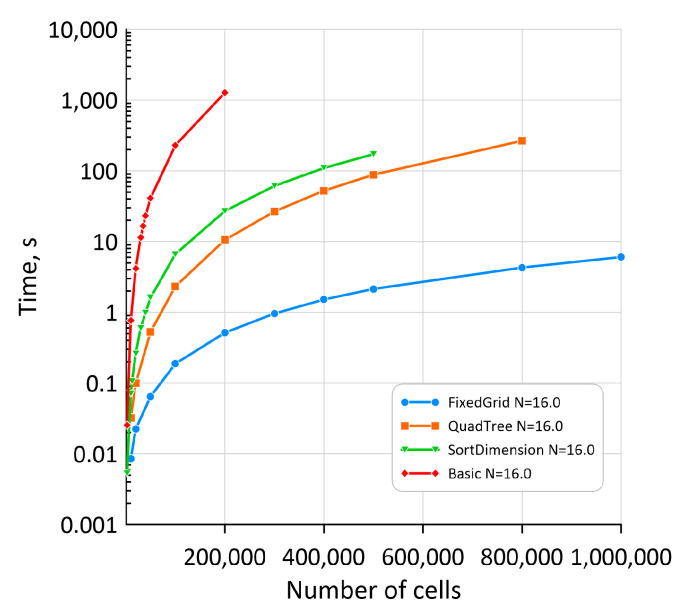
The time overhead of various neighbour search algorithms.

As presented in Figure 7, the latter approaches applied to the RCA unconstrained grain growth effectively eliminated the problem of the neighbourhood search computational overhead in the developed model. The concept of grouping cells in fixed grid containers (so-called buckets) allowed for the handling of 1,000,000 RCA cells in a magnitude of seconds.

**Figure 8:**
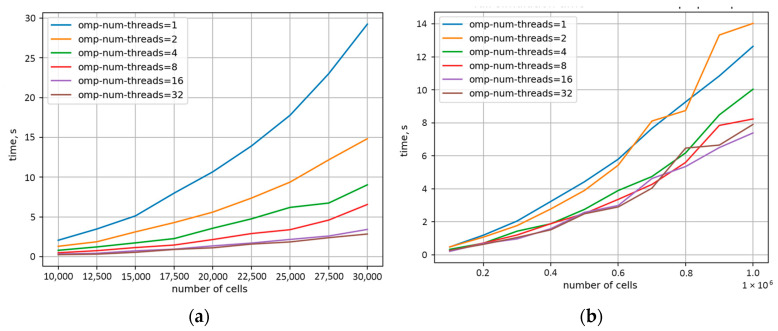
Computation time reduction for (**a**) basic and (**b**) bucket-based parallel algorithms implemented to increase the omp thread number.

Therefore, the current research selected the bucket-based approach in the RCA neighbour evaluation step for additional parallelisation with the OpenMP (omp) standard to further improve the code computational efficiency. During parallelisation, the classical formulation #pragma omp parallel for was used. Different schedule mechanisms were also tested, and a runtime scheme was selected as the best: schedule (runtime). The second approach used for parallelisation was based on the #pragma omp task. Such parallelisation schemes were also applied to the basic neighbour search algorithm as a lower-bound case for comparison purposes. From an algorithmic point of view, it was necessary to independently parallelise the preparatory function responsible for arranging subsequent calculations and the main execution function responsible for calculations in each time step. In the preparation step (prep) of the bucket-based algorithm, the cells located throughout the space were appropriately assigned to the buckets within which they were located. With that, in the execution phase (step), only the nearest buckets were analysed to determine the neighbours of an investigated RCA cell. The results of the OpenMP implementation are summarised in Figure 8. The analysis was performed on a platform with 16 physical cores (2xXeon E5-2620 v4) and hyperthreading leading to 32 OpenMP threads.

**Figure 9:**
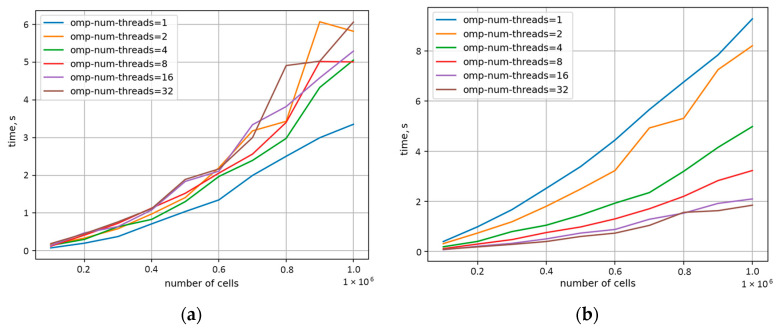
Computation time reduction for the bucket-based algorithm in the (**a**) preparatory step and (**b**) execution step for increasing the omp thread number.

As shown in Figure 8a, parallelisation of the basic search algorithm already allows for a significant reduction in the calculation time with respect to the sequential version; however, even after parallelisation, the algorithm continued to perform significantly worse than the sequential versions of earlier investigated algorithms in Figure 7. On the other hand, the parallel version of the bucket-based search algorithm provided the results approx. 40% faster for eight threads. At the same time, above this thread level, a further increase in computational resources does not provide any additional benefits in computational cost reduction, as seen in Figure 8b. This behaviour is due to the code dependencies in the preparatory step of the method, as shown in Figure 9a, where parallelisation even worsened the computation time. Therefore, in such a case, new parallelisation concepts should be developed for the preparatory step, or it should be run in a sequential manner. On the contrary, the second (execution) step after parallelisation revealed clear improvement, as presented in Figure 9b.

Despite some identified constraints, the developed algorithmic solutions in the random cellular automata code for unconstrained grain growth significantly reduced the execution times and allowed further development of the complex dynamic recrystallisation model.

### 2.3. RCAFE Dynamic Recrystallisation Model

As mentioned, dynamic recrystallisation is controlled by two main phenomena: new grains’ nucleation and subsequent growth. The model of nucleation proposed by Ding and Guo [26] was used in the current research to tackle the first phenomenon. The model assumes that there is a relationship between the strain rate $\dot{\epsilon }$ and the nucleation rate $\dot{n}$ [27,28]. Additionally, the impact of temperature is also considered as follows:

(8) $$\dot{n}\left(\dot{\epsilon },\;T\right)={\dot{\epsilon }}^{m}\exp\left(-\frac{Q_{act}}{RT}\right)$$

where *C* is a constant, $Q_{act}$ is the activation energy, *R* is the gas constant, and *m* is the strain rate sensitivity exponent.

Nucleation can occur only in the RCA cells located on grain boundaries when the dislocation density in the given cell exceeds the critical value ($\rho_{i,j}>\rho_{crit}$). The critical dislocation density is determined with the Roberts and Ahlblom formula [26]:

(9) $$\rho_{crit}={\left(\frac{20\gamma_{k}\dot{\epsilon }}{3blM_{k}\tau^{2}}\right)}^{\frac{1}{3}}$$

where $\gamma_{k}$ is the grain boundary energy, $M_{k}$ is the grain boundary mobility, and τ is the energy required for dislocation movement.

The dislocation density is updated in each RCA cell based on the FE calculated equivalent stress value in the corresponding integration points with the mean dislocation density formula:

(10) $$\bar{\rho }=\frac{1}{\rho_{0}}{\left(\frac{\sigma_{i}}{a_{6}Gb}\right)}^{2}$$

where *G* is the shear modulus, *b* is Burger’s vector, and $\sigma_{i}$ is the equivalent stress value from the FE simulation step.

Then, after nucleation, the driving force for the growth of new, recrystallised grains comes from the difference in dislocation density between new grains and the surrounding, deformed area. The grain growth model assumes that the growth velocity is obtained as a multiplication of the driving force $F_{i}$, and grain boundary mobility *M* as follows:

(11) $$V_{i}=MF_{i}$$

The mobility term is calculated as follows [11]:

(12) $$M=\frac{\delta D_{ob}b}{KT}\exp\left(-\frac{Q_{b}}{RT}\right)$$

where δ is the grain boundary thickness, $Q_{b}$ is the grain boundary diffusion activation energy, *K* is the Boltzman constant, and $D_{ob}$ is the grain boundary self-diffusion coefficient.

At the same time, the driving force for grain boundary movement is expressed as follows:

(13) $$F=\alpha \mu b^{2}\Delta \rho =2\alpha \tau \left(\rho_{m}-\rho_{i}\right)$$

where α is the coefficient, $\rho_{i}$ is the dislocation density of recrystallised grains, and $\rho_{m}$ is the dislocation density of the surrounding area.

Finally, the velocity of grain growth is used to determine the recrystallisation fraction in each RCA cell as follows:

(14) $$\Delta \alpha_{i}=\frac{V_{i}\Delta t}{l}$$

where Δt is the step time and *l* is the distance between the considered unrecrystallised and the neighbouring recrystallised cells in the RCA space.

If a recrystallised fraction in the RCA cell exceeds a value of one, then the cell becomes recrystallised. At the same time, it becomes a part of the neighbouring recrystallised grain and its dislocation density is set to the initial value prior to deformation $\rho_{init}$.

To allow coupling between the RCA and FE models, each RCA cell additionally contains information on its position in the computational domain and its physical size. With that, information from the FE mesh can be directly mapped into the corresponding RCA cloud of points in each simulation time step. As a result, the RCAFE model is established and can be used for DRX simulations under high-temperature deformation.

## 3. Results

The final coupling between the developed in-house RCA model and the commercial Abaqus 2023 FE software was performed with the user subroutine option. The developed flow stress for Fe30Ni limited to hardening and recovery phenomena was used during the FE simulations, while the RCA model considered the influence of DRX.

**Figure 10:**
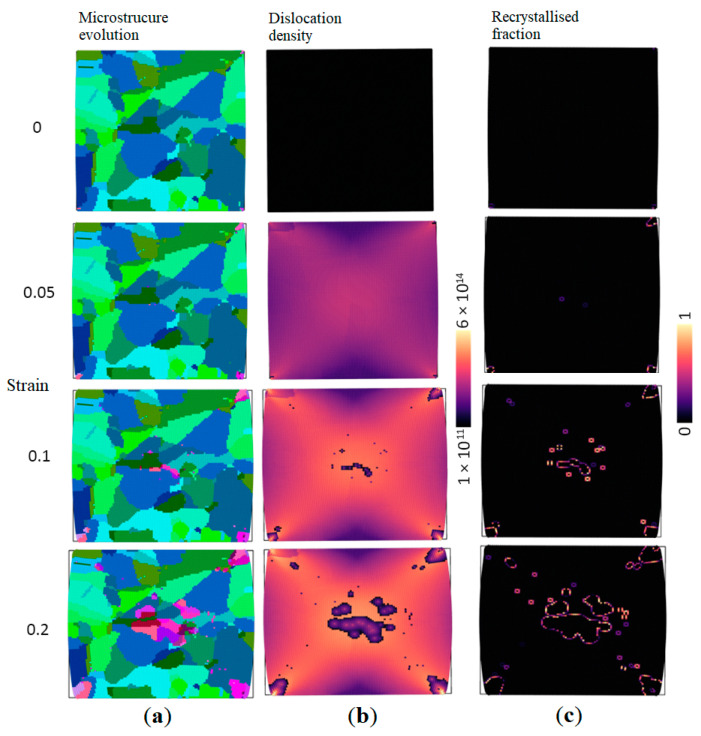
Examples of results from the developed RCAFE model: (**a**) material morphology evolution, (**b**) recrystallisation volume fraction, and (**c**) dislocation density evolution (visualisation with OVITO [29]).

An example of a simulation outcome from the developed RCAFE DRX model for the analysis of the plane strain compression test is presented in Figure 10.

As presented in Figure 10, the developed full-field model can explicitly predict the nucleation and subsequent growth of new grains driven by the stored energy in the dislocation fields. The geometrical changes in the computational domain due to deformation are also accounted. However, before the RCAFE DRX model can be used for practical case study simulations, a robustness analysis has to be performed to prove its reliability.

## 4. Discussion

### RCAFE DRX Model Robustness Analysis

**Figure 11:**
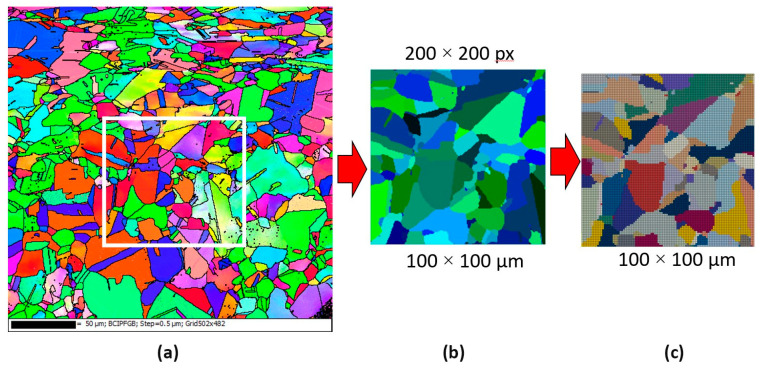
Development of the initial digital microstructure morphology for the DRX simulation: (**a**) EBSD map, (**b**) extracted digital microstructure morphology model, and (**c**) finite element discretisation of the model.

First, the mesh sensitivity study was performed, and various levels of mesh discretisation were assigned to the model. The digital representation model of the microstructure prior to deformation was directly extracted from the as-received Fe30Ni material and used as input for subsequent RCAFE simulations. The electron back-scattered diffraction map with a step size of 0.5 µm was obtained with the EBSD detector (EDAX, Mahwah, NJ, USA) within the scanning electron microscope (FEI, Hillsboro, OR, USA) and directly translated into the required digital format, as presented in Figure 11. A research area of 100 × 100 µm was used for RCAFE modelling purposes.

**Figure 12:**
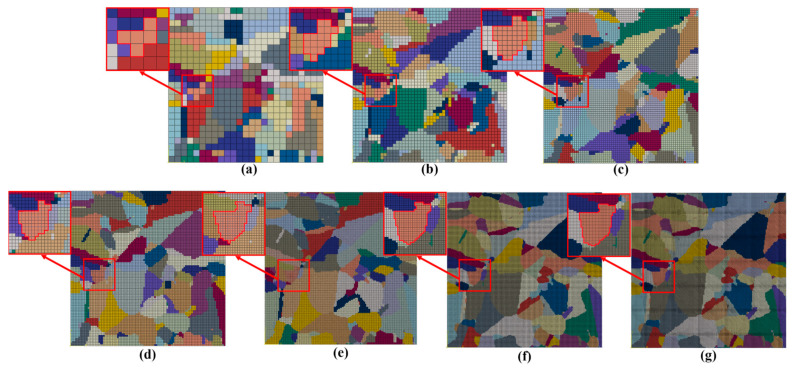
The starting FE meshes employed in the numerical simulation of plane strain compression: (**a**) 25 × 25, (**b**) 50 × 50, (**c**) 75 × 75, (**d**) 100 × 100, (**e**) 150 × 150, (**f**) 200 × 200, and (**g**) 250 × 250 elements with emphasis on a selected grain in red square.

In the RCAFE model, particular finite elements are assigned to individual grains and are characterised by slightly varied material properties to reflect the differences in the crystallographic orientation. The number of RCA cells directly correlates with the number of FE elements used during the investigation and increases from 25 × 25 to 250 × 250, as shown in Figure 12.

**Figure 13:**
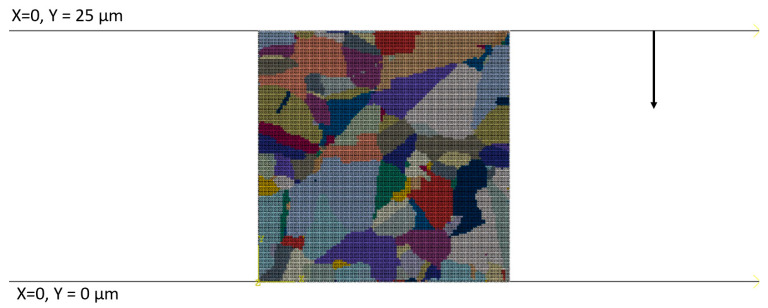
Boundary conditions of numerical simulation of plane strain compression.

Each of the presented models was deformed under plane strain conditions at 1000 °C and a strain rate of 1/s (Figure 13). The neighbourhood radius in the parallel bucket-based search algorithm was set to ensure an average of eight RCA cells in the area of the investigated cell. Data transfer from the FE to the RCA model was unidirectional at this research stage.

**Figure 14:**
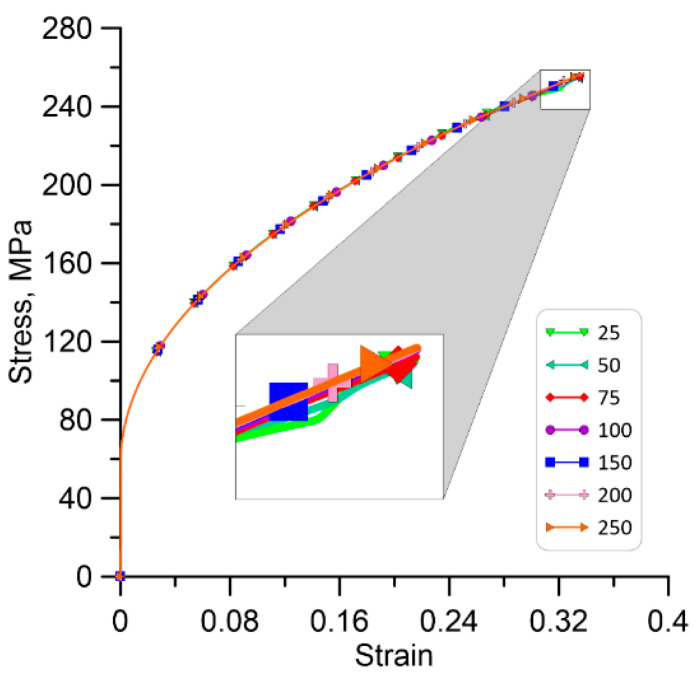
Homogenised stress–strain responses for the different mesh sizes used during the simulation.

**Figure 15:**
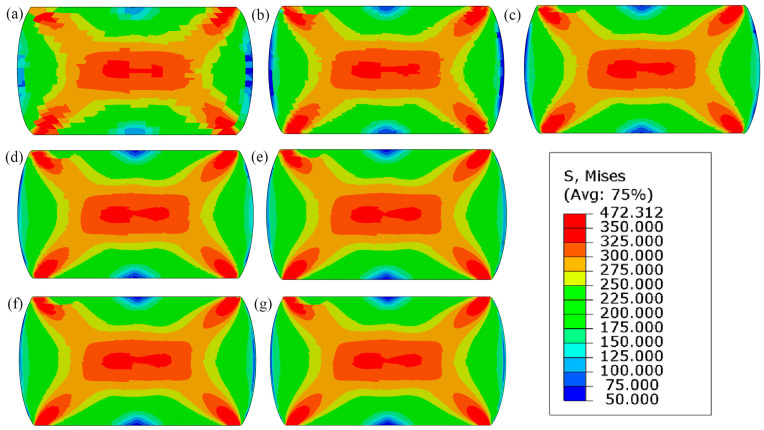
Equivalent plastic stress field at the end of loading for increasing discretisation levels: (**a**) 25 × 25, (**b**) 50 × 50, (**c**) 75 × 75, (**d**) 100 × 100, (**e**) 150 × 150, (**f**) 200 × 200, and (**g**) 250 × 250 finite elements.

**Figure 16:**
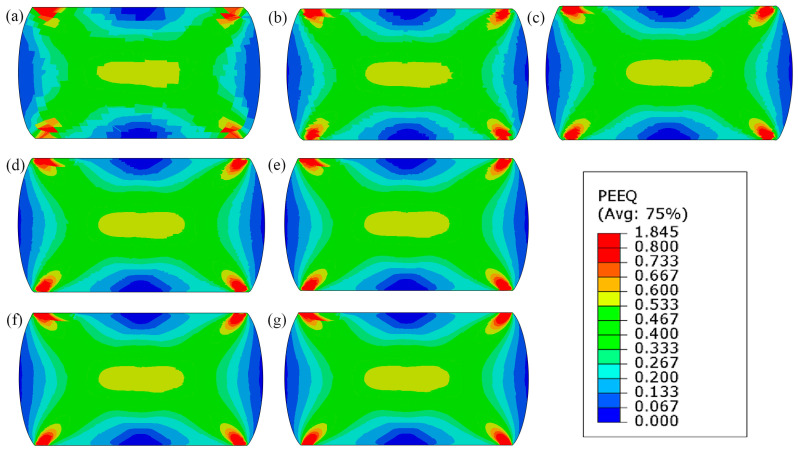
Equivalent plastic strain field at the end of loading for increasing discretisation levels: (**a**) 25 × 25, (**b**) 50 × 50, (**c**) 75 × 75, (**d**) 100 × 100, (**e**) 150 × 150, (**f**) 200 × 200, and (**g**) 250 × 250 finite elements.

Figure 14, Figure 15 and Figure 16 shows the qualitative and quantitative findings of the mesh sensitivity analysis conducted through a series of FE simulations.

As can be seen in Figure 14, the general material response in the FE simulation remains consistent, despite notable variations in mesh densities. Nonetheless, small disturbances are noticed in the local equivalent stress and strain distributions (Figure 15 and Figure 16). As a result, a minimum discretisation level of approximately 75 × 75 elements per 100 × 100 µm is recommended for the FE model to ensure consistent outcomes.

**Figure 17:**
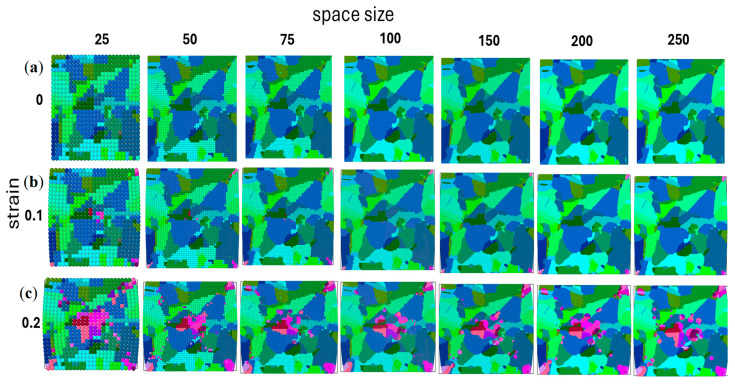
Material morphology for an increasing number of RCA cells in the computational space at the (**a**) initial step and (**b**) 0.1 and (**c**) 0.2 strain levels.

Figure 17 displays the corresponding RCA results and illustrates variations in recrystallisation morphologies at different stages of the simulation. Given the stochastic components within the RCA model, each iteration was executed three times to capture a broader range of responses.

**Figure 18:**
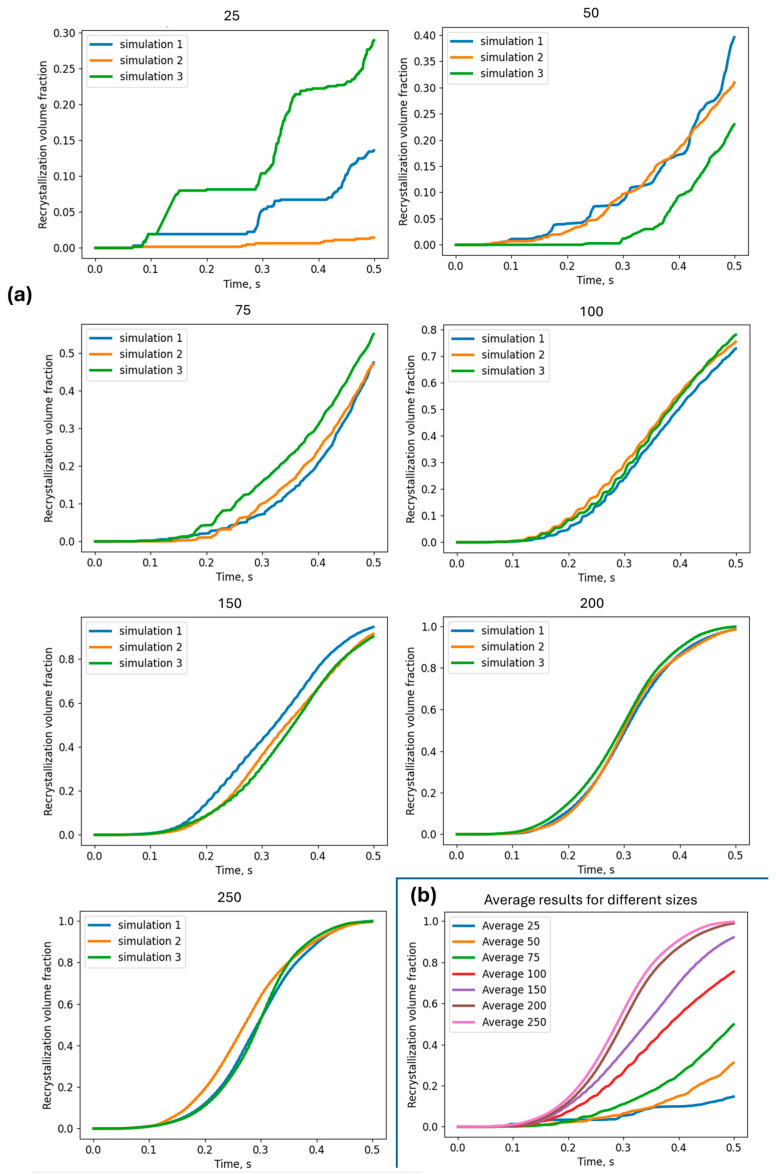
Comparison of (**a**) recrystallisation volume fractions for multiple runs of simulations with an increasing number of cells in space and (**b**) averaged responses.

In this case, the convergence of the RCA model requires substantially larger mesh sizes. As can be noticed, starting from the level of 200 × 200 RCA cells in the computational space, grain boundaries become smoother, and grain evolution is quite similar in the investigated microstructures. Detailed results in the form of the recrystallisation volume fractions of multiple runs of simulation are presented in Figure 18. A final comparison of the averaged recrystallisation volume fractions is also presented in that figure.

**Figure 19:**
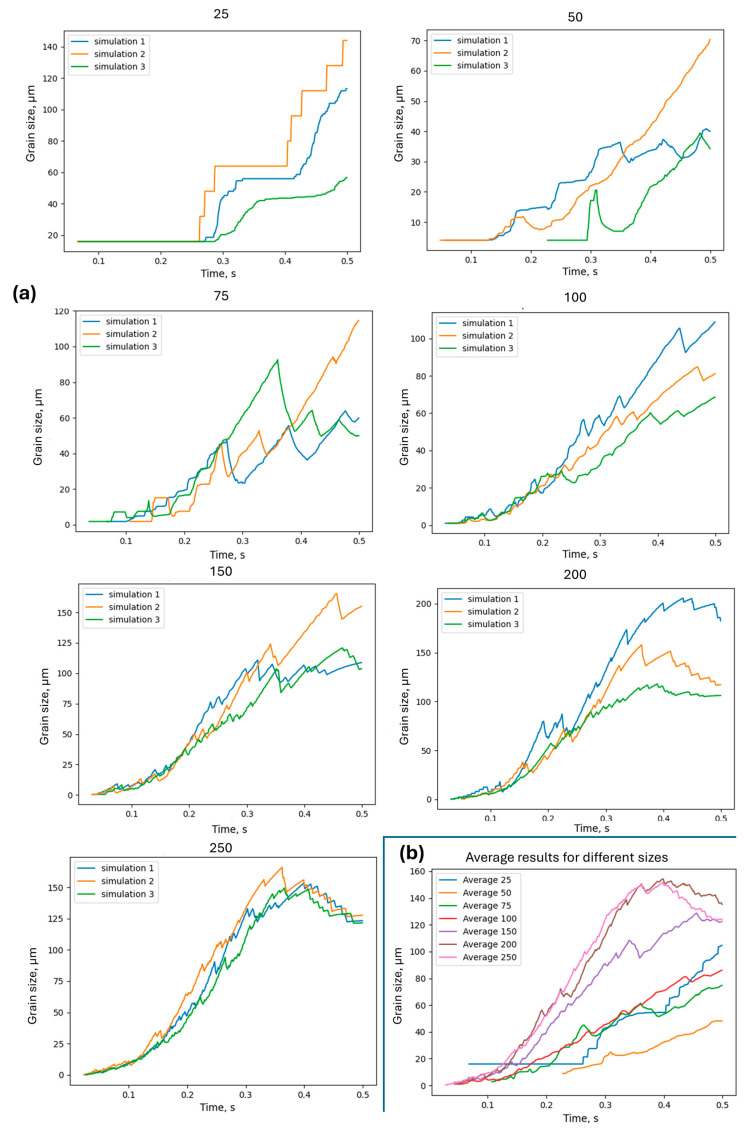
Comparison of (**a**) the average grain size for multiple runs of simulations with an increasing number of cells in space and (**b**) averaged responses.

Additionally, an interesting model prediction of the average grain size evolution during DRX can be observed in Figure 19.

The low mesh density clearly affects the evolution of the average grain size during the DRX. However, again from the level of 200 × 200 RCA cells in the computational space, the results start to converge.

**Figure 20:**
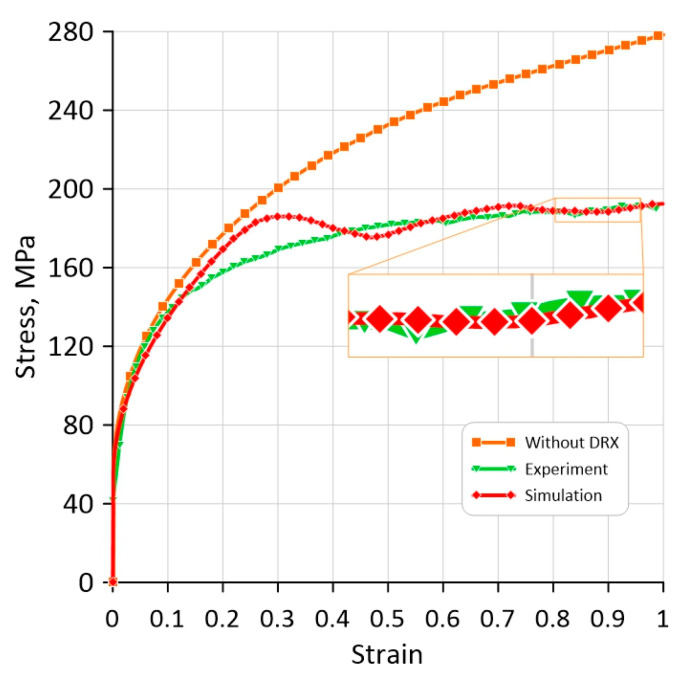
Comparison of final model results after the parameter identification procedure.

Therefore, as presented, the minimal threshold for initial cell size can be evaluated, and geometrical issues related to the classical CA approach can be eliminated with the random CA variant. Finally, the flow stress evolution obtained with such an RCAFE model is presented in Figure 20 against the corresponding experimentally measured values from Figure 6. The theoretical flow stress evolution, considering hardening and recovery only, has also been added for comparison purposes.

The presented results are a milestone in developing the fully coupled RCAFE model that will solve the limitations of the cellular automata method in capturing computational space deformation. At the same time, it is proven that with the appropriate implementation of the RCA code, the computational effort will not be a constraint for the 3D simulations.

## 5. Conclusions

The development and implementation of a coupled random cellular automata finite element approach for full-field modelling of dynamic recrystallisation was presented in this paper. It was shown that the RCAFE model can directly consider the influence of geometrical changes in the microstructure during large plastic deformation on recrystallisation progress. Therefore, the main limitation of the classical cellular automata method based on a regular grid of CA cells was mitigated. However, such a coupled RCAFE model requires cooperation between two methods involving data transfer between interacting computational domains, which may be time-consuming. Therefore, to maintain the attractiveness of the RCAFE method, particular attention has to be paid to code efficiency during the implementation stages.

At this stage of the model development, the following sets of detailed conclusions and guidelines can be formulated:

- The use of reliable experimental input data is critical for numerical model development stages, and therefore, the inverse analysis technique is recommended for data interpretation as it can take into account the influence of process heterogeneities on the final outcome;
- The development of an appropriate neighbour selection algorithm is a critical step from the RCA model simulation time reduction point of view. The bucket-based concept proved its capabilities in the RCA applications;
- Parallelisation with the OpenMP standard provides additional capabilities in computational time reduction but has to be applied based on a series of efficiency tests to identify the limits of its applicability.
- The developed DXR RCAFE model can properly capture major mechanisms of dynamic recrystallisation and can be the basis for further improvements to incorporate other phenomena during nucleation and grain growth;
- Despite the stochastic elements in the RCA model that introduce some variations in the simulation results, the model with a certain computational space size provides repeatable results;
- Both the recrystallisation kinetics and the microstructural morphology of finer meshes can be adequately reproduced during the simulation, but the RCA part of the model determines the minimum mesh size.

The next step of the research on developing the DRX RCAFE model will focus on a final mesh sensitivity study in a fully coupled model, where the RCA predictions affect the FE simulation in each time step and vice versa. This step is required prior to the final model parameter identification and validation, which will be the last step of RCAFE model development.

## Figures and Tables

**Table 1:** Chemical composition of the Fe30Ni alloy.

C	Si	Mn	P	S	Mo	Ni	Al	Cu	V	W	Fe
0.0638	0.187	1.67	0.016	0.0172	1.59	30	0.01	0.027	0.02	0.06	Bal

## Data Availability

The data that support the findings of this study are available from the corresponding author, Ł.M., upon reasonable request.
